# Study on lockage safety of LNG-fueled ships based on FSA

**DOI:** 10.1371/journal.pone.0174448

**Published:** 2017-04-24

**Authors:** Pengfei Lv, Yuan Zhuang, Jian Deng, Wei Su

**Affiliations:** School of Navigation, Wuhan University of Technology, Wuhan, Hubei, China; Seoul National University, REPUBLIC OF KOREA

## Abstract

In the present study, formal safety assessment (FSA) is introduced to investigate lockage safety of LNG-fueled ships. Risk sources during lockage of LNG-fueled ships in four typical scenarios, namely, navigation between two dams, lockage, anchorage, and fueling, are identified, and studied in combination with fundamental leakage probabilities of various components of LNG storage tanks, and simulation results of accident consequences. Some suggestions for lockage safety management of LNG-fueled ships are then proposed. The present research results have certain practical significance for promoting applications of LNG-fueled ships along Chuanjiang River and in Three Gorges Reservoir Region.

## Introduction

With growing demand for energy in China, the introduction of the liquefied natural gas (LNG) can contribute to optimization of energy structures, and effectively solve energy supply problems. Moreover, the LNG can play an important role in many aspects. Recently, the use of the LNG as a ship power fuel showed favorable economic and environmental benefits. Various provinces and cities along Yangtze River started to gradually launch pilot projects involving LNG-fueled ships. As many large LNG receiving stations and wharfs are successively put into operation along Yangtze River, the number of LNG ships started to see sustainable growth. Since LNG-fueled ships have higher safety risks than ordinary cargo ships, due to its specially properties, pool fire, vapour clouds, rollover and several other types of hazards are existing, navigation safety of LNG ships appears to be particularly important.

LNG-fueled ships started to be used along Bokna Fjord in Norway in recent years. Due to the special geographic position and pivotal role of Three Gorges ship lock, the lockage of LNG-fueled ships have not yet been allowed in order to ensure the lock’s safety of operation and safety of passengers and cargoes on other ships. Due to particular hazards of LNG fuels such as inflammability and susceptibility to explosion, special and extremely strict safety management should be conducted on LNG-fueled ships. Although LNG-fueled ships have not yet become extensively popular, scholars started to conduct related research. Adachi *et al*. applied the discount cash flow method (DCFM) to analyze the economy of a 9300 TEU container ship fueled by the LNG [1]. The result shows that LNG-fueled container ships using a low speed diesel directly coupled propulsion system are not only more attractive as an investment being environment friendly, but also more economical than Tier III complied oil-fueled containerships. Lee *et al*. systematically analyzed fire risks of fuel systems of LNG-fueled ships, and performed dynamic simulations using actual navigation data [2]. Wang *et al*. systematically reviewed literature on LNG-fueled ships and their development prospects [3]. Zhang *et al*. conducted simulations on leakage diffusion behavior of LNG-fueled ships using Safer Trace software. By fitting three-dimensional simulation data on LNG leakage and diffusion behavior at different wind velocities and under different atmospheric stability conditions, they derived the relationships among the leakage amount of LNG, gas concentration, and diffusion distance [4]. Andersen *et al*. investigated the cost effectiveness of LNG-fueled ships [5]. Additionally, some scholars conducted simulations on the diffusion of LNG, and their results provide data support for the quantitative analysis of LNG-fueled ship accidents [6–11].

The International Maritime Organization (IMO) provided the *International Code for the Construction and Equipment of Ships Carrying Liquefied Gases in Bulk* (IGC Code) and *The Interim Guidelines on Safety for Natural Gas-Fueled Engine Installation for Ships* [12–13]. American Bureau of Standards (ABS) also provided relevant regulations and guidelines, including *ABS Guide for the Propulsion Systems of Gas Carriers* and *ABS Guide for the Propulsion and Auxiliary Systems for Gas Fueled Ships* [14]. The International Association of Classification Societies (IACS) made uniform requirements for control and safety systems of dual-fuel diesel engines. However, there is still lack of research on safety assessment and regulations of LNG-fueled ships under some special navigation conditions, such as LNG fueling, berthing and so on. With the development of China’s national economy, a great number of LNG-fueled ships were put into operation, where safety management must be considered when they pass through Three Gorges ship lock. The present study can provide references for investigation of feasibility of lockage of LNG-fueled ships through Three Gorges ship lock, and have certain practical significance for enhancing the safety level of inland navigation of LNG-fueled ships. In this paper, risk events causing loss of lives are mainly considered.

In this article, a theoretical system of formal safety assessment (FSA) is first introduced in Section 1.The identification of the risks of LNG-fueled ships in four typical scenarios, namely, navigation between two dams, lockage, anchorage for berthing and unberthing, and fueling, are described in detail in Section 2. In Section 3, risk assessment of the lockage of LNG-fueled ships is conducted from two aspects, probability and consequences, and the assessment results are compared with related standards for analysis. Finally, Section 4 proposes related suggestions for the lockage safety of LNG-fueled ships based on the assessment results.

## Section 1 theoretical system of formal safety assessment (FSA)

### Introduction to FSA

The FSA is a comprehensive, structured, systematic and feasible analysis method that can be used for providing reasonable and practicable risk controls in engineering technologies and operation management [15]. The FSA adopts normalized five steps, and comprehensively assesses projects related to ships’ design, inspection, operation and navigation, so as to effectively improve the maritime safety degree in many aspects such as life at sea, crew’s health, marine environment and resources of ships, goods and possessions.

Compared to other safety assessment methods, the assessment procedures of the FSA are more formal and reasonable. It is a posteriori and anticipatory formal safety assessment method that provides accurate and reliable assessment results which can be directly used for a long period of time. Besides, the existing safety assessment methods mainly adopt qualitative methods for analysis and assessment. Using the FSA, we can calculate produced expenses and benefits, and conduct related analysis based on risk assessment and control schemes, and then conduct in-detail quantitative analysis and assessment. Moreover, we can make reasonable decisions on safety requirements in accordance with the actual ratio of expenses to benefits [16].

### Procedures of FSA

**Fig 1:**
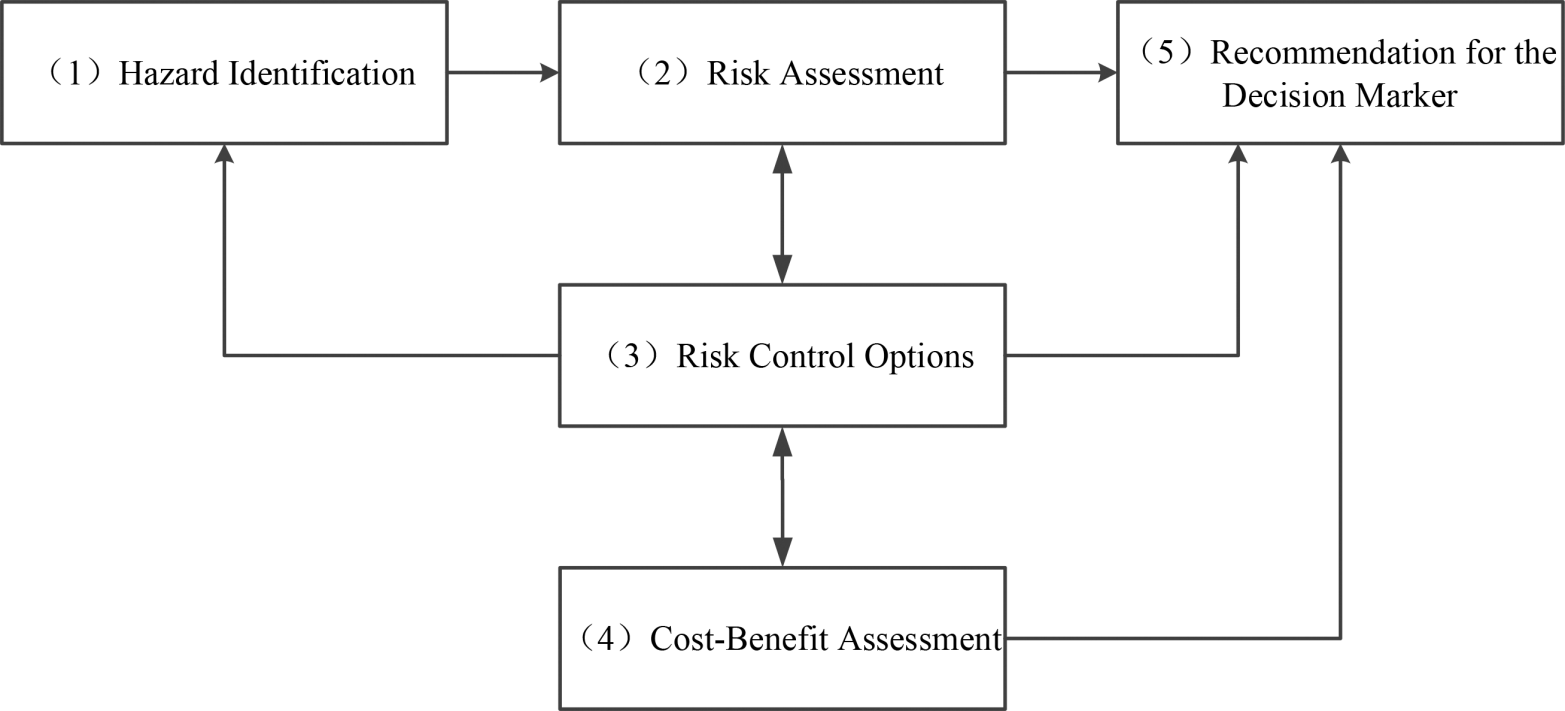
Flow chart of FSA method.

As shown in **Fig 1**, the FSA includes five formal assessment procedures, namely, hazard identification, risk assessment, risk control schemes, expense and benefit assessment, and decision-making suggestions.

1. Hazard Identification. As the initial step of the FSA, it aims to identify all possible hazards in the defined assessment system project, and then make a corresponding list according to different hazard levels, so as to further analyze primary hazards.
2. Risk Assessment. Based on the determination of the existence and objective distribution of risks, various factors that affect the risk degrees are analyzed and sorted to find high and key risk factors. Moreover, the relationship between accident occurrence and consequences is discussed, so that the existing standards or regulations can be revised, some new standards or regulation can be made, and the occurrence of risks can be reduced.
3. Risk Control Options. Based on hazard identification and risk assessment results, some targeted measures for risk reduction are proposed, and then specific and feasible risk control schemes are introduced, including the formulation and revision of some standards and regulations.
4. Cost-benefit Assessment. This step refers to the estimation and assessment of costs and benefits when each risk control option is adopted. Accordingly, the risk control options are ranked to propose reasonable decision-making suggestions in the following step.
5. Recommendation for the Decision Maker. In this step, reasonable decision-making recommendations are proposed based on the comparison and ranking of hazards and potential causes, as well as cost-benefit evaluations when different risk control options are used.

The FSA mainly exhibits three basic flow forms: 1-2-5, 1-2-3-5 and 1-2-3-4-5 [17]. Generally, the formulation of risk control schemes and assessment of expenses and benefits should be analyzed by various professionals in related fields, otherwise, the calculations are difficult. This is the primary reason for omitting the third and fourth steps in the implementation of the FSA. In the present study, the FSA using the flow form 1-2-5 is adopted for investigation of the lockage safety of LNG-fueled ships in Three Gorges ship lock.

## Section 2 identification of risks during lockage of LNG-fueled ships

In the present study, risk identification is conducted in four typical scenarios during navigation of LNG-fueled ships, namely, navigation between two dams, lockage, anchorage for berthing and unberthing and fueling. The following risk sources are mainly obtained from literature reviews.

(1) Navigation between two dams

Multiple factors can affect the navigation safety of LNG-fueled ships in the dam area, mainly including related parameters and the navigation environment of the dam area, such as the flow, wind, visibility, aids to navigation, and ship traffic flow. Accidents mainly include striking a reef or buildings, and collisions of ships. The causes of accidents are primarily related to the ships’ safety, external navigation environment, and crews’ behavior [18]. The consequences of accidents can be classified into ordinary collision accidents and secondary hazards.

(2) Lockage

In general, ships pass through the lock at relatively low velocities, thus there is a low risk of the occurrence of collision accidents. The emission of the boil-off gas (BOG) is the main risk of fire hazards of LNG-fueled ships. The traffic flow in the area of Three Gorges Dam along Yangtze River is high, in particular, all ships pass through the lock in the way of uniform marshalling, and, therefore, small distances between ships in the lock chamber might cause fire on other ships in the chamber when a fire accident occurs on one ship, causing severe secondary hazards. Accordingly, fire accidents are the primary risk of the lockage of ships. The main causes are described below. Ships generally wait a long time when queuing up before the lock. Due to the effects of a continuous hot weather, the pressure in the LNG storage tank increases, the pressure relief valve is opened, and then the BOGs of the LNG are discharged from the permeability pipe, which can cause a fire accident when exposed to an open flame. Additionally, due to poor safety awareness, some crew may smoke in LNG-fueled ships or nearby danger zones, which can cause fire accidents [19].

If a fire accident occurs during the lockage of a ship, fire can spread rapidly, simultaneously imposing serious impact on other ships in the lock, resulting in fearful consequences.

(3) Anchorage for berthing and unberthing

Since the operations in the anchorage area are busy, and many problems such as human factors, ships, environment and management exist, the collisions between ships and contact damage accidents may occur during navigation in this area, leading to great harm to related crews, ships and buildings [20]. For LNG-fueled ships, the primary risks in the anchorage area are collisions and contact damages.

(4) Fueling

On account of human disoperation, substandard quality of equipment, wear failures, and other reasons, the fueling software and flanged joints may fail, leading to the leakage and diffusion of the LNG. The primary risks during the LNG fueling process are pipeline breaks and over-fueling. The causes of accidents mainly include leakage in the flanged joints of fueling and air-returning pipelines, dangers in the ground lines and fixed lines connecting LNG tank cars, rupture of LNG flexible hoses, failure of LNG fueling arm, incorrect temperatures in the fueling tank and injecting tank, and failure or absence of quick-disconnection devices in emergency circumstances [21].

In view of differences between LNG-fueled ships and ordinary ships, accidents of the lockage of LNG-fueled ships may cause deaths and huge economic losses. Fire accidents in the engine and living quarters of LNG ships are likely to result in failure of insulating layers of LNG tanks, causing fires or explosions, and thereby damages to the exposed crew. According to the above risk identification analysis results, the accident risks during the navigation and lockage of LNG-fueled ships are investigated and listed in Table 1.

**Table 1:** Accident risks of LNG-fueled ships during navigation and lockage processes.

State of the ships	Dangerous accidents	Accident risks	
		Failure mode	Consequences
Navigation between two dams	Striking a reef	Leakage from storage tanks, pipelines and valves	Fire, explosion, freezing injuries, cold brittle failure
	Collision between ships	Leakage from storage tanks, pipelines and valves	Fire, explosion, freezing injuries, cold brittle failure
Lockage	Fire accident	Ignition of gases emitted from the overpressure safety valve	Fire, explosion
	Damages to the ship	Leakage from storage tanks, pipelines and valves	Fire, explosion, freezing injuries, cold brittle failure
Fueling	Failure of LNG fueling pipelines	Leakage from pipelines	Fire, explosion, freezing injuries, cold brittle failure
	Over-fueling	Ruptures of storage tanks	Fire, explosion
Anchorage	Collision between ships	Leakage from storage tanks, pipelines and valves	Fire, explosion, freezing injuries, cold brittle failure
	Damages to the ship	Leakage from storage tanks, pipelines and valves	Fire, explosion, freezing injuries, cold brittle failure

## Section 3 risk assessment of lockage of LNG-fueled ships

Based on the above risk identification results, accidents of LNG-fueled ships during navigation and lockage are mainly connected with leakage of LNG storage tanks. There are two typical leakage modes, namely, instantaneous and continuous leakage. The major factors that affect the intensity of leakage are the leakage hole’s size and shape. The size of hole greatly affects the accident scenario and range of consequences. Generally, three types of leakage holes, namely, small, medium-sized and large holes, are considered [22–24], having diameters of 5 mm, 50 mm and 100 mm, respectively. In the present study, the wind velocity relative to the ship is set as 3 m/s, and the external temperature is set as 20℃.

According to the above leakage modes and scenarios of LNG-fueled ships, we analyze possible accident scenarios after the occurrence of LNG leakage by considering hazardous characteristics of the LNG, operating conditions, and matter states during storage, gasification and gas-mixing processes of LNG-fueled ships.

### Quantitative risk assessment theory and related standards

Quantitative risk assessment, also known as probability risk assessment (PRA), refers to quantitative analysis of the probability of system or equipment failure, and severity of failures, making accurate descriptions of system risks. The PRA can quantitatively determine the hazard level of the evaluated object. In the present study, individual risks are calculated. An individual risk is defined as the annual occurrence probability of individual deaths at a certain position without any safeguard measures induced by all potential accidents:

$$\text{R}(x,\;y)=\sum_{\text{t}=1}^{\text{n}}f_{\text{t}}{\text{V}}_{\text{t}}(x,y)$$ (1)

Where R(*x*, *y*) denotes the individual risk at position (x, y); *f*_t_ denotes the occurrence probability of the *t*_th_ accident; V_t_(*x*, *y*) denotes the probability of individual death at position (x, y) induced by the *t*_th_ accident; and n denotes the number of accidents. In this formula, *f*_t_ represents probability of individual risk, and V_t_(*x*, *y*) represents consequence of individual risk.

According to the No. 40 Order issued by the State Administration of Work Safety [25], individual risks at important targets and sensitive sites around enterprises with dangerous chemicals should satisfy the admissible risk standards shown in Table 2.

**Table 2:** Individual risk standards in China.

Important targets and sensitive sites around enterprises with dangerous chemicals	Admissible risks
1. High-sensitivity sites (such as schools, hospitals, kindergartens and nursing homes)	3×10^−7^
2. Important targets (such as Party and government offices, military areas and culture relic protection sites)	
3. Special sites having high-density population (such as large stadiums and large transportation junctions)	
4. Residential buildings having high-density population (such as residential areas, hotels and holiday villages)	1×10^−6^
5. Public buildings having high-density population (such as offices, markets, restaurants and entertainment venues)	

### LNG leakage probability

According to the analysis results on LNG leakage modes, system leakages of LNG storage tanks mainly include storage tank leakage, pipeline leakage, and the leakage from some accessories such as valves.

By referring to basic leakage frequencies of different components listed in *The Fundamental Methods Based on Risk Inspection* (SYT 6714–2008) [26], the leakage probabilities of LNG storage tanks, pipelines and valves under different failure modes are calculated, as shown in Table 3.

**Table 3:** LNG accident probabilities under different failure modes.

Component type		Failure mode	Probability of accident occurrence F_i_(per year)	
			Flash fire	Vapor cloud explosion
LNG storage tank	15m^3^/20m^3^	Small-hole leakage	1.20×10^−9^	8.00×10^−10^
		Medium-hole leakage	6.00×10^−8^	4.00×10^−8^
		Large-hole leakage	6.00×10^−9^	4.00×10^−9^
Pipeline	DN40	Small-hole leakage	3.60×10^−8^	2.40×10^−8^
		Medium-hole leakage	7.20×10^−9^	4.80×10^−9^
	DN25	Small-hole leakage	6.00×10^−8^	4.00×10^−8^
		Rupture of all pipes	6.00×10^−9^	4.00×10^−9^
	DN10	Small-hole leakage	1.20×10^−7^	8.00×10^−8^
		Rupture of all pipes	3.60×10^−9^	2.40×10^−9^
Valve	DN40	Small-hole leakage	3.34×10^−8^	2.22×10^−8^
		Medium-hole leakage	4.14×10^−9^	2.76×10^−9^
	DN10	Small-hole leakage	3.34×10^−8^	2.22×10^−8^
		Complete rupture	1.80×10^−9^	1.20×10^−9^

#### Simulations of accident consequences of LNG-fueled ships

**Fig 2:**
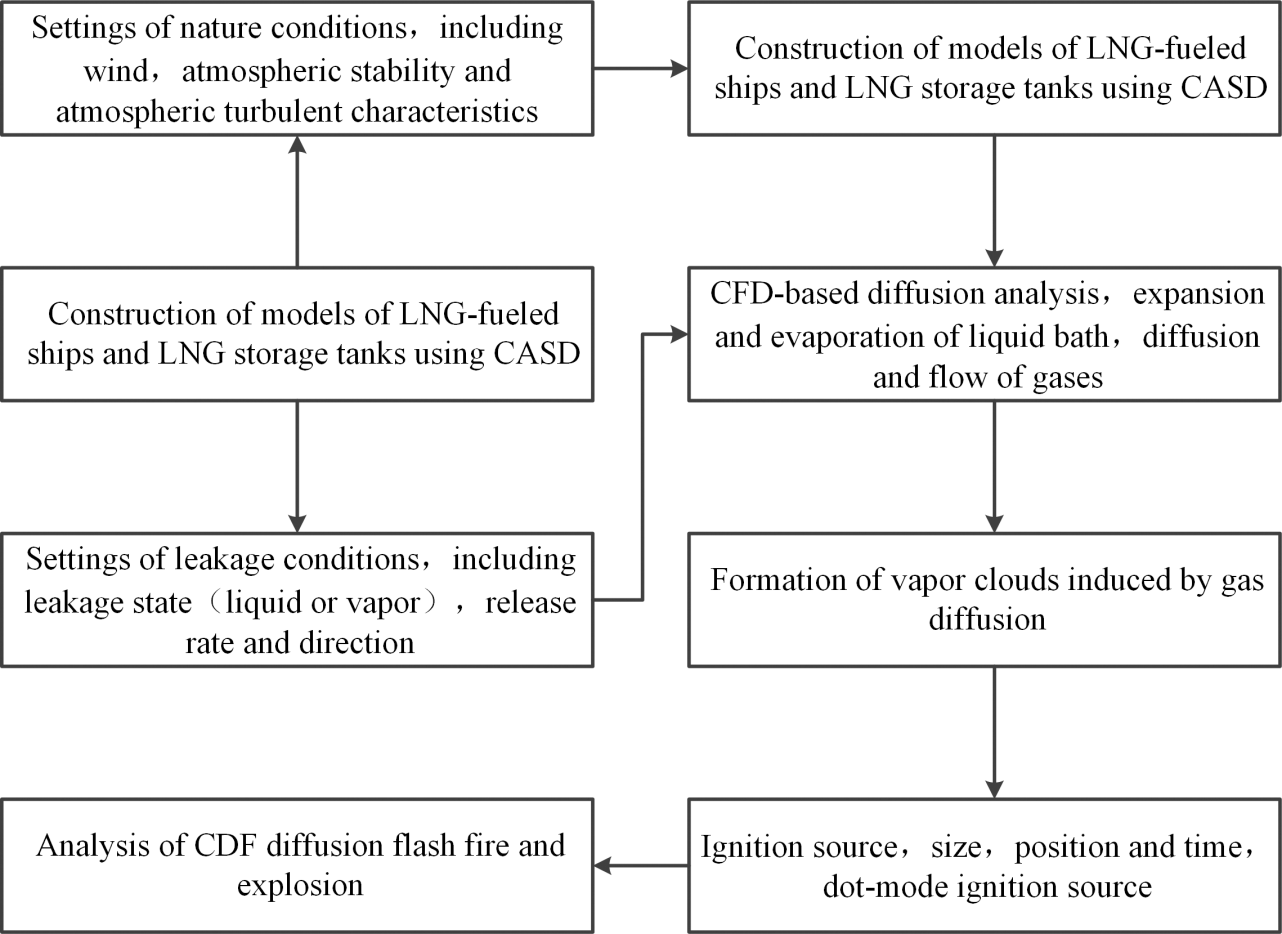
Computational process using FLACS.

In the present study, gas diffusion and vapor cloud explosion (VCE) are simulated using the FLACS software. The FLACS is a three-dimensional software set for computational fluid dynamics (CFD), which can accurately simulate gas diffusion states and the propagation of explosive shock waves. **Fig 2** illustrates the computational process using the FLACS.

The ‘Red Sun 166’ is the first certificated LNG-fueled ship in China, whose reconstruction technology is qualified through inspection. Since the operation time is long, test data is abundant. This makes it representative for LNG-fueled ships at the present stage. Therefore, the ‘Red Sun 166’ was selected as an example for simulations and calculations in the present study.

According to discussion results of experts from Changjiang Maritime Safety Administration, DN40 pipeline leakage, DN10 pipeline leakage, DN40 valve leakage and DN10 valve leakage may appear on the ‘Red Sun 166’. The main accident types are flash fire and the VCE. The evaporation time of the LNG is set as the typical diffusion time of continuous leakage, 10 min, which is recommended by the World Bank, and the evaporation rate of the LNG is set as 0.049 kg/m^2^·s. Based on the radius of the flash-fire damage range and ship’s size presented in related reports, the possible casualties induced by flash fire and the VCE are estimated through experts’ discussions, as shown in Table 4 and Table 5.

**Table 4:** Consequences of flash-fire accidents under different failure modes.

		Diffusion radius of LNG at LEL (m)	Flash-fire range (m)	Possible number of casualties
Storage tank,15m^3^	Small	3.43	3.24	2
	Medium	55.3	20.48	6
	Large	75.2	56.15	10
DN40 pipeline	Small	3.88	3.55	2
	Medium	20.06	12.44	4
DN10 pipeline	Small	3.12	2.98	1
	Complete	12.88	3.62	2
DN40 valve	Small	3.46	3.42	2
	Medium	20.86	12.35	4
DN10 valve	Small	3.33	3.23	2
	Complete	12.76	3.56	2

**Table 5:** Consequences of VCE accidents under different failure modes.

		Radius of slight injuries (m)		Radius of moderate injuries (m)	Radius of serious injuries (m)	Possible number of casualties
Storage tank,15m^3^	Small	No occurrence				
	Medium	9.64	9.55		9.33	4
	Large	67.14	61.71		51.62	10
Pipeline linkage		No occurrence				
Valve linkage		No occurrence				

As shown in Table 4, the greater the leakage diameter of the LNG storage tank, the larger the damage range. Therefore, some appropriate measures should be adopted to control failure modes of LNG storage tanks, so that medium and large leakage and complete rapture accidents can be avoided, and damages induced by flash-fire accidents can be alleviated.

### Calculation of overall leakage risks of LNG-fueled ships

According to Eq (1), in combination with various probabilities and simulated accident consequences, the overall comprehensive risk value of LNG leakage under different failure modes was calculated, as shown in Table 6.

**Table 6:** Flash fire/VCE risk under different failure modes.

		Leakage probability	Flash-fire probability	VCE probability	Comprehensive risk value
Storage tank, 15m^3^	Small	1.00×10^−7^	0.012	N/A	2.40×10^−9^
	Medium	5.00×10^−6^	0.03	0.008	9.00×10^−7^
	Large	5.00×10^−7^	0.03	0.008	1.50×10^−7^
DN40 pipeline	Small	3.00×10^−6^	0.012	N/A	7.20×10^−8^
	Medium	6.00×10^−7^	0.012	N/A	2.88×10^−8^
DN10 pipeline	Small	1.00×10^−5^	0.012	N/A	1.20×10^−7^
	Complete	3.00×10^−7^	0.012	N/A	7.20×10^−9^
DN40 valve	Small	2.78×10^−6^	0.012	N/A	6.67×10^−8^
	Medium	3.45×10^−7^	0.012	N/A	1.66×10^−8^
DN10 valve	Small	2.78×10^−6^	0.012	N/A	6.67×10^−8^
	Complete	1.50×10^−7^	0.012	N/A	3.60×10^−9^
Overall risk value					1.63×10^−6^

### Calculation results and comparisons

According to the calculated results presented in Table 6, the overall risk value of flash-fire and VCE accidents induced by LNG leakage is 1.63×10^−6^. This means that the above risks can result in 1.63×10^−6^ casualty accidents each year.

The overall risk of LNG leakage is higher than the corresponding risk standard at high-sensitivity or special sites having high-density population (3×10^−7^)[27], which demonstrates that the workers of Three Gorges ship lock face high risks when an LNG-fueled ship passes through the lock. In other words, LNG-fueled ships should not be allowed to pass through Three Gorges ship lock at the present stage.

## Section 4 suggestions for lockage safety management of LNG-fueled ships

There are mainly two ways to control risks of LNG-fueled ships during lockage, namely, reducing the accident occurrence probability and controlling induced consequences. In the present study, by fully considering the specifics of the lockage of LNG-fueled ships, safety management suggestions for LNG-fueled ships in different typical scenarios, namely, navigation between two dams, lockage and anchorage, are proposed.

(1) Navigation between two dams

1) LNG storage tanks should be installed and arranged in strict accordance with the *Rule for Natural Gas Fueled Ships* formulated by China Classification Society (CCS), and the emission of the BOG during navigation through Three Gorges ship lock should also be controlled by taking related measures.

2) During navigation between dams, ships can be fueled using a hybrid of oil and gas until oil and gas pipelines are inspected to be qualified.

3) When a main engine failure of an LNG-fueled ship occurs, it should be reported to the VTS center in time, to select an appropriate water area for anchoring.

4) During navigation in the dam area, the crew should keep frequent lookouts and navigate carefully at an appropriate safety speed. If necessary, some effective anti-collision measures should be taken to prevent close quarter situations.

5) After entering the dam area, the ship’s position should be reasonably adjusted according to the wind flow at that time, and the ship should keep a safety distance from buildings to avoid ship collision accidents.

6) During navigation in the flood season, attention should be paid to the control of yaw angle in accordance with the wind and flow-pressure angle of the wind flow at that time, together with continuous use of the rudder and steerage to effectively control ship’s deviation, so that the ship can safely pass through the dam.

7) Supervision of the navigation safety in the dam area should be strengthened. Especially in some key regions and accident-prone areas, regional supervision should be strengthened, and special coastal patrol vessels should be provided to convoy LNG-fueled ships.

(2) Lockage

1) In view of emissions and accumulation of the BOG, it is suggested that LNG-fueled ships should be separated when passing through the lock, i.e. the appearance of two or more LNG-fueled ships in the lock chamber should be avoided.

2) Ships should use oil-fueled modes to reduce effects induced by instability of LNG fuel systems.

3) Using real time monitoring of ships’ operating states in the lock, the lock management departments can communicate with crews so that they can find and deal with existing but hidden safety dangers in time.

4) The completeness and correctness of ships’ registration information when passing through the lock should be checked, so that the possibility of concealed or false declaration of ship information is reduced, and thus the lockage safety is enhanced.

5) LNG-fueled ships should not pass through the lock together with ships caring dangerous goods or passenger ships.

(3) Anchorage

1) During berthing and unberthing operations, LNG-fueled ships should use oil-fueled modes to reduce effects induced by instability of LNG fuel systems.

2) Ships’ speeds should be reasonably controlled in accordance with the requirements of the *Code for Loads of Port Engineering*.

3) Currently, management can be strengthened according to practical conditions of LNG-fueled ships during anchoring and waiting for lockage. When the number of LNG-fueled ships reaches a certain value, the administration department of Three Gorges lock should consider the feasibility of setting special anchorage areas for this type of ships, and strengthen fire protection in such areas.

Besides, in the view of the specifics of the navigation environment in the area of Three Gorges Dam and danger during fueling, it is suggested that the fueling of LNG-fueled ships should be forbidden in Three Gorges Reservoir Region.

(4) Safety management of LNG-fueled ships

1) On the premise of adoption of appropriate safety precautions in many aspects, such as the design and layout of storage tanks, ship management, and training of crew, it is suggested that inland LNG-fueled ships with non-dangerous goods should be managed as general ships with non-dangerous goods.

2) Based on the proposed safety management recommendations in the present study, it is suggested that related departments should further refine safety precautions for the lockage of LNG-fueled ships in the following aspects: the equipment of ships’ safety facilities, ships’ daily safety management, safety management of fueling, safety management in special regions, maritime safety management, emergency management, and crew management.

## Conclusions

Using the framework of the FSA, this article comprehensively analyzes the risks of LNG-fueled ships when passing through Three Gorges ship lock. Using leakage probabilities of various components of LNG storage tanks and simulation results of accident consequences, the risks of LNG-fueled ships in four typical scenarios, namely, navigation between two dams, lockage, anchorage, and fueling, are analyzed. It is concluded that LNG-fueled ships should not pass through Three Gorges ship lock at the present stage. In addition, some safety management suggestions for the lockage of LNG-fueled ships a provided according to hazard identification and risk assessment results.

## Supporting information

S1 File Parameter symbol. (DOCX)
