# Supplementary material for: Study on lockage safety of LNG-fueled ships based on FSA
Source: PLoS One. 2017 Apr 24;12(4):e0174448. doi: 10.1371/journal.pone.0174448 (PMC5402973; doi:10.1371/journal.pone.0174448)
Supplement: S1 File — (DOCX) [file pone.0174448.s001.docx]

 $\text{R(}x\text{，}y\text{)}$ the individual risk at position (x, y);

$f_{\text{t}}$ the probability of individual risk;

 the consequence of individual risk;
